# Recurrent Subclinical Tuberculosis Among Antiretroviral Therapy–Accessing Participants: Incidence, Clinical Course, and Outcomes^[Author-notes ciac185-FM1]^

**DOI:** 10.1093/cid/ciac185

**Published:** 2022-03-05

**Authors:** Kogieleum Naidoo, Mikaila C Moodley, Razia Hassan-Moosa, Navisha Dookie, Nonhlanhla Yende-Zuma, Rubeshan Perumal, Halima Dawood, Nomonde R Mvelase, Barun Mathema, Salim Abdool Karim

**Affiliations:** Centre for the AIDS Programme of Research in South Africa, Nelson R Mandela School of Medicine, College of Health Sciences, University of KwaZulu-Natal, Durban, South Africa; South African Medical Research Council-CAPRISA HIV-TB Pathogenesis and Treatment Research Unit, Doris Duke Medical Research Institute, University of KwaZulu-Natal, Durban, South Africa; Centre for the AIDS Programme of Research in South Africa, Nelson R Mandela School of Medicine, College of Health Sciences, University of KwaZulu-Natal, Durban, South Africa; Centre for the AIDS Programme of Research in South Africa, Nelson R Mandela School of Medicine, College of Health Sciences, University of KwaZulu-Natal, Durban, South Africa; South African Medical Research Council-CAPRISA HIV-TB Pathogenesis and Treatment Research Unit, Doris Duke Medical Research Institute, University of KwaZulu-Natal, Durban, South Africa; Centre for the AIDS Programme of Research in South Africa, Nelson R Mandela School of Medicine, College of Health Sciences, University of KwaZulu-Natal, Durban, South Africa; South African Medical Research Council-CAPRISA HIV-TB Pathogenesis and Treatment Research Unit, Doris Duke Medical Research Institute, University of KwaZulu-Natal, Durban, South Africa; Centre for the AIDS Programme of Research in South Africa, Nelson R Mandela School of Medicine, College of Health Sciences, University of KwaZulu-Natal, Durban, South Africa; South African Medical Research Council-CAPRISA HIV-TB Pathogenesis and Treatment Research Unit, Doris Duke Medical Research Institute, University of KwaZulu-Natal, Durban, South Africa; Centre for the AIDS Programme of Research in South Africa, Nelson R Mandela School of Medicine, College of Health Sciences, University of KwaZulu-Natal, Durban, South Africa; South African Medical Research Council-CAPRISA HIV-TB Pathogenesis and Treatment Research Unit, Doris Duke Medical Research Institute, University of KwaZulu-Natal, Durban, South Africa; Division of Pulmonology, Inkosi Albert Luthuli Central Hospital, Nelson R. Mandela School of Medicine, University of KwaZulu-Natal, Durban, South Africa; Centre for the AIDS Programme of Research in South Africa, Nelson R Mandela School of Medicine, College of Health Sciences, University of KwaZulu-Natal, Durban, South Africa; Department of Internal Medicine, Infectious Diseases Unit, Grey’s Hospital, University of KwaZulu-Natal, South Africa; Department of Medical Microbiology, KwaZulu-Natal Academic Complex, National Health Laboratory Service, Durban, South Africa; Department of Medical Microbiology, School of Laboratory Medicine and Medical Sciences, College of Health Sciences, University of KwaZulu-Natal, Durban, South Africa; Department of Epidemiology, Mailman School of Public Health, Columbia University, New York, New York, USA; Centre for the AIDS Programme of Research in South Africa, Nelson R Mandela School of Medicine, College of Health Sciences, University of KwaZulu-Natal, Durban, South Africa; South African Medical Research Council-CAPRISA HIV-TB Pathogenesis and Treatment Research Unit, Doris Duke Medical Research Institute, University of KwaZulu-Natal, Durban, South Africa; Department of Epidemiology, Mailman School of Public Health, Columbia University, New York, New York, USA

**Keywords:** subclinical TB, HIV, prevalence, incidence, clinical TB

## Abstract

**Background:**

Undiagnosed asymptomatic subclinical tuberculosis (TB) remains a significant threat to global TB control, accounting for a substantial proportion of cases among people living with human immunodeficiency virus (HIV)/AIDS (PLWHA). We determined incidence, progression, and outcomes of subclinical TB in antiretroviral therapy (ART)–accessing PLWHA with known previous TB in South Africa.

**Methods:**

A total of 402 adult PLWHA previously treated for TB were enrolled in the prospective Centre for the AIDS Programme of Research in South Africa TRuTH (TB Recurrence Upon TB and HIV treatment) Study. Participants were screened for TB with quarterly clinical and bacteriologic evaluation and biannual chest radiographs over 36 months. Those with suspected or confirmed TB were referred to the National TB Programme. Participants received HIV services, including ART. Incidence rate of TB was estimated using Poisson regression and descriptive statistical analyses summarized data.

**Results:**

A total of 48 of 402 (11.9%) bacteriologically confirmed incident recurrent TB cases were identified, comprising 17 of 48 (35.4%) subclinical TB cases and 31 of 48 (64.5%) clinical TB cases. Age, sex, and body mass index were similar among subclinical, clinical, and no TB groups. Incidence rates (95% Confidence Interval [CI]) of recurrent TB overall, in clinical and subclinical TB groups were 2.3 (1.7-3.0), 1.5 (1.1-2.2), and 0.9 (0.5-1.4) per 100 person-years, respectively. In the subclinical TB group, 14 of 17 (82.4%) were diagnosed by TB culture only, 11 of 17 (64.7%) received TB treatment, and 6 of 17 (35.3%) resolved TB spontaneously.

**Conclusions:**

High incidence rates of recurrent subclinical TB in PLWHA highlight inadequacies of symptom-based TB screening in high TB–HIV burden settings.

## Teaser

Undiagnosed asymptomatic subclinical tuberculosis (TB) threatens global TB control. Similarly high incidence rates of recurrent clinical and subclinical TB in people living with human immunodeficiency virus/AIDS highlight inadequacies of symptom-based TB screening in high-burden settings.

**Clinical Trials Registration**. NCT01539005.

Globally, tuberculosis (TB) mortality remains unacceptably high, with 1.4 million deaths each year [1]. The World Health Organization’s ambitious End TB strategy focuses on early TB diagnosis and treatment of symptomatic individuals to achieve a 90% reduction in TB incidence by 2035 [2]. Despite growing evidence of an asymptomatic subclinical TB phase, referral into the TB diagnostic and care continuum relies on symptom-based TB screening tools [3–6]. Subclinical TB does not present with typical TB-related symptoms of cough, fever, night sweats, or weight loss, often going undetected with the currently recommended TB screening tools [7–9]. These seemingly well individuals harbor viable TB bacilli, representing a pool of potentially infectious individuals who may be missed by existing TB prevention efforts and consequently pose a hidden threat to global TB control ^[2, 10]^.

Deeper understanding of TB pathogenesis highlights distinct pathophysiological phases within the TB disease spectrum [5]. Diagnostic criteria for subclinical TB include absence of typical TB-related symptoms coupled with identification of viable *Mycobacterium tuberculosis* (*M.tb*) bacilli on sputum mycobacterial culture [5, 11]. There are currently no accurate estimates of subclinical TB incidence and prevalence rates. Published population-representative TB prevalence studies have postulated higher rates of subclinical TB compared with annual TB case notifications. Using the average prevalence-to-notification ratio of 2:1, the global prevalence of subclinical TB can be estimated at 7 million cases, with varying estimates among people living with human immunodeficiency virus (HIV)/AIDS (PLWHA) [2, 6, 12, 13].

Existing TB diagnostic tests used in resource-limited settings, such as Gene Xpert *Mycobacterium tuberculosis*/rifampin (Cepheid), chest radiography, tuberculin skin test, and sputum microscopy, present unique challenges in detecting subclinical TB [2, 13]. Abnormal chest radiography is not consistently included in the subclinical TB case definition, and TB smear microscopy is often inefficient in diagnosing subclinical TB in PLWHA with paucibacillary disease [5, 7]. Sputum culture remains the gold standard to confirm TB diagnosis in participants with subclinical TB [14].

Additionally, risk factors for progression of subclinical to clinical TB in endemic populations are poorly described. Studies propose that asymptomatic immunosuppressed subclinical TB PLWHA with radiographic lesions may rapidly progress from subclinical to clinical TB, highlighting the need for early triage for TB diagnostic testing in those at high risk for progression to clinical TB [15, 16]. Further research is required to comprehensively understand the pathogenesis of subclinical TB, establish rates of progression to clinical TB, and determine the outcomes of subclinical TB in PLWHA.

Published data from cohort studies evaluating the natural history of subclinical TB are limited. We report incidence, progression, and outcomes of subclinical TB among antiretroviral therapy (ART)–accessing PLWHA in a TB-endemic South African setting.

## METHODS

### Study Population

Adult TB–HIV-coinfected patients on ART who successfully completed treatment for pulmonary TB in Centre for the AIDS Programme of Research in South Africa (CAPRISA) TB–HIV treatment trials were enrolled into the TB Recurrence Upon TB and HIV treatment (TRuTH) Study. This prospective longitudinal cohort study was conducted between 2009 and 2013 (SANCTR DOH-27-0909-3040, Biomedical Research Ethics Committee of the University of KwaZulu-Natal, BF051/09). A total of 402 ambulant participants were enrolled and followed up prospectively over 36 months for incident TB at the CAPRISA eThekwini Research Site in Durban, KwaZulu-Natal.

### Study Procedures and Data Collection

At quarterly follow-up visits, participants’ medical history, evaluation of recent TB contact information, TB symptom screening, and clinical examination findings were assessed. Chest radiography was conducted biannually, irrespective of TB symptoms, or when clinically indicated. TB mycobacteriology was processed at the University of KwaZulu-Natal Medical Microbiology Laboratory. At each quarterly visit, participants produced 2 induced sputum samples following ultrasonic nebulization with normal (0.9%) saline. Sputum was sent to a TB laboratory for acid-fast bacilli (AFB) smear (auramine and Ziel Nielson), liquid and solid culture, and drug susceptibility testing. Cluster of differentiation 4 (CD4) count, flow cytometry (FACS Calibur, Becton Dickinson), HIV RNA (Cobas Amplicor HIV-1 Monitor, version 1.5, Roche), and safety blood testing were done at a local laboratory. Participants with bacteriologically confirmed TB met the definition for incident TB. Participants diagnosed with TB were referred to the National TB Control Programme for treatment. Participants with no symptoms or signs of clinical TB were offered TB preventive therapy (TPT) when this became standard of care nationally. During follow-up, all participants had access to 6-monthly ART safety and efficacy laboratory monitoring. Detailed TB history, site of TB, clinical course, and outcome data were collected for each incident TB episode (Supplementary Figure 1).

The following definitions were adopted for this analysis: subclinical TB, asymptomatic TB, that is, a negative TB symptom screen with identification of TB by sputum smear or culture, and clinical TB, symptomatic TB, that is, a positive TB symptom screen and identification of TB by sputum smear or culture [5]. Definitions of TB treatment outcome followed prevailing guidelines [17].

### Statistical Analyses

Data were summarized using medians with interquartile range (IQR), mean with standard deviation, and frequencies with percentages. Poisson regression with person-years as an offset was used to calculate incidence rate and 95% confidence intervals (CIs). Statistical analysis was performed using SAS version 9.4 (SAS Institute, Cary, NC).

## RESULTS

### Recurrent TB Diagnosis

**Figure 1.**
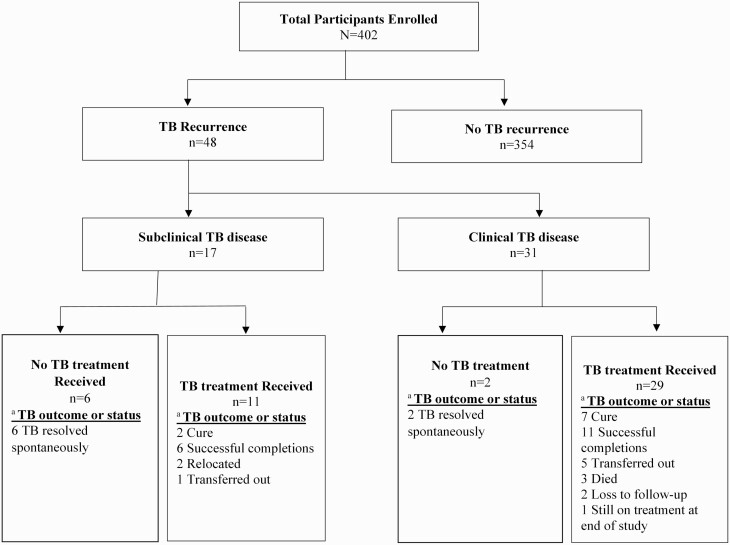
Recurrent clinical and subclinical TB among people living with human immunodeficiency virus/AIDS on antiretroviral therapy previously successfully treated for TB. ^a^All treatment outcomes are defined per World Health Organization definitions and South African national TB treatment guidelines as follows: cure, a participant whose baseline smear or culture was positive at the start of treatment and is smear- or culture-negative in the last month of treatment and on at least 1 previous occasion 30 days prior; treatment completed, a participant whose baseline smear or culture was positive at the start of treatment and has completed treatment but does not have a negative smear or culture in the last month of treatment and on at least 1 previous occasion more than 30 days prior. The smear examination may not have been done or the results may not be available at the end of treatment. Abbreviation: TB, tuberculosis.

Overall, among 402 participants, 11.9% (48 of 402) developed bacteriologically confirmed TB, with subclinical TB identified in 4.2% (17 of 402). Among incident TB cases, subclinical and clinical TB comprised 35.4% (17 of 48) and 64.5% (31 of 48), respectively (Figure 1). Median time to diagnosis from last TB outcome was 41 months (IQR, 30–54) overall and 34 months (IQR, 26–43) and 45 months (IQR, 32–57) for those with subclinical and clinical TB, respectively. The incident rate of TB was 2.3 (95% CI: 1.7–3.0), 1.5 (95% CI: 1.1–2.2), and 0.9 (95% CI: .5–1.4) per 100 person-years for clinical and subclinical TB, respectively.

### Baseline Clinical and Demographic Characteristics

Baseline clinical and demographic characteristics were compared among participants with subclinical TB, clinical TB, and no recurrent TB (Table 1). The proportion of subclinical and clinical TB participants with an undetectable viral load was 70.6% and 61.3% at baseline and 94.1% and 70.0% at recurrent TB diagnosis, respectively (Table 1). Median time on ART was 3.3 years (IQR, 2.3–4.0) and 4.3 years (IQR, 3.0–5.1) for the subclinical and clinical TB groups, respectively (Table 1).

**Table 1.** Clinical and Demographic Characteristics of Enrolled Participants at Baseline and at Tuberculosis Recurrence

Characteristic	Category	All, N = 402 (%)	Subclinical TB, n = 17 (%)	Clinical TB, n = 31 (%)	No TB, n = 354 (%)
Mean age (SD), years		38.2 (9.1)	37.0 (6.3)	35.0 (9.0)	38.6 (9.2)
Gender, n (%)	Male	186 (46.3)	9 (52.9)	15 (48.4)	162 (45.8)
	Female	216 (53.7)	8 (47.1)	16 (51.6)	192 (54.2)
Median CD4 (IQR), cells/mm^3^		453 (329–643)	330 (270–502)	367 (188–689)	465 (344–645)
Viral load, n (%), copies/mL	Undetectable (<50)	333 (82.8)	12 (70.6)	19 (61.3)	302 (85.3)
	Detectable	69 (17.2)	5 (29.4)	12 (38.7)	52 (14.7)
Mean weight (SD), kg		68.1 (13.3)	61.8 (9.5)	65.1 (12.2)	68.7 (13.5)
Mean body mass index (SD), kg/m^2^		26.4 (5.6)	24.0 (3.7)	25.2 (5.6)	26.6 (5.7)
Lung cavities, n (%)	None	396 (98.5)	17 (100.0)	31 (100.0)	348 (98.3)
	One	6 (1.5)	0	0	6 (1.7)
Lung infiltrates, n (%)	None	326 (81.1)	14 (82.4)	19 (61.3)	293 (82.8)
	One	14 (3.5)	1 (5.9)	1 (3.2)	12 (3.4)
	Bilateral	62 (15.4)	2 (11.8)	11 (35.5)	49 (13.8)
Lung adenopathy, n (%)	None	395 (98.3)	16 (94.1)	31 (100.0)	348 (98.3)
	One	3 (0.7)	0	0	3 (0.8)
	Bilateral	4 (1.0)	1 (5.9)	0	3 (0.8)
Lung pleural, n (%)	None	394 (98.0)	17 (100.0)	29 (93.5)	348 (98.3)
	One	7 (1.7)	0	2 (6.5)	5 (1.4)
	Bilateral	1 (0.2)	0	0	1 (0.3)
Characteristics at TB or before recurrence^a^					
Median time on antiretroviral therapy (IQR), years		3.8 (2.8–4.7)	3.3 (2.3–4.0)	4.3 (3.0–5.1)	
Median CD4 + IQR		405 (251–679)	369 (291–589)	439 (241–711)	
Viral load (copies/mL),^b^ n (%)	Undetectable (<50)	37 (78.7)	16 (94.1)	21 (70.0)	
	Detectable	10 (21.3)	1 (5.9)	9 (30.0)	
Sputum culture positive^c^			16	27	
Liquid media			16	18	
Solid media	CFU^d^ ≥10		5	9	
	CFU ≤10		4	1	
Time to sputum culture positivity^e^ (range in days)			8–19	3–18	

Abbreviations: CD, cluster of differentiation; CFU, colony-forming unit; IQR, interquartile range; SD, standard deviation; TB, tuberculosis.

^a^Characteristics recorded within 5 months before recurrence.

^b^Missing baseline data. One participant had a missing pre-recurrence viral load.

^c^Six participants with clinical TB were diagnosed on chest X ray (CXR), 3 with CXR only and 3 with both CXR and TB smear positivity.

^d^CFU counts were not known for 8 culture-positive clinical TB participants.

^e^Time to sputum culture positivity was missing for 2 subclinical TB participants.

**Figure 2.**
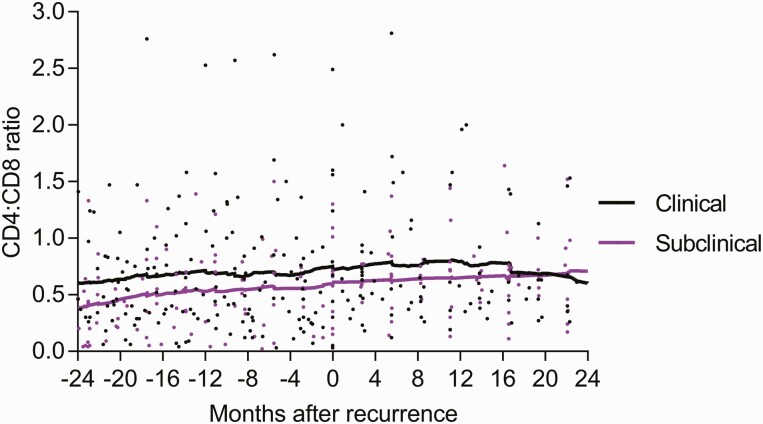
CD4:CD8 ratio pre- and post-recurrent subclinical and clinical tuberculosis (TB) diagnosis. Abbreviation: CD, cluster of differentiation.

**Figure 3.**
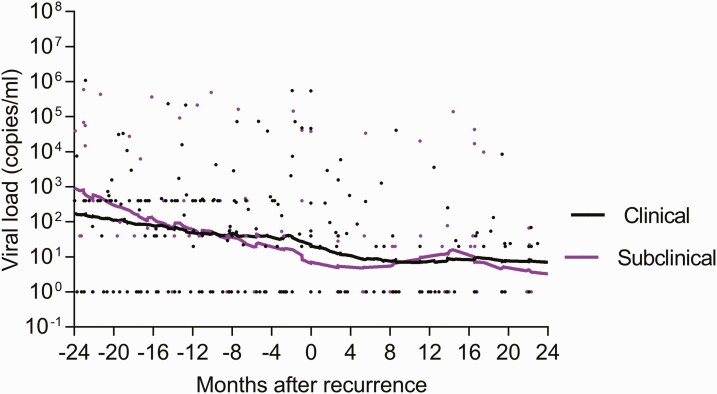
Viral load pre- and post-recurrent subclinical and clinical tuberculosis (TB) diagnosis.

Time to symptom onset from bacteriologic diagnosis of recurrent TB ranged from 1 to 8 months among subclinical TB participants who progressed to clinical TB (Tables 2 and 3). Perturbation in plasma viral load and CD4:CD8 cell count ratio was observed 4 months before and after TB recurrence (Figures 2 and 3).

**Table 2.** Clinical Progression and Outcomes Among Treated Subclinical Tuberculosis Participants

Participant	Months to Symptom Onset from Bacteriologic Diagnosis of Recurrent TB^a^	Months from Recurrent TB Diagnosis to Treatment Start	Time to Culture Conversion from Recurrent TB Diagnosis, Months^b,c^	Months on Antiretroviral Therapy at TB Recurrence	Clinical Features of TB Disease Progression^d^	Relative % Change in Weight^e^
1	2.3	20.3	16.4	56.6	Unexplained mild, nonproductive cough	+0.5
2	5.0	4.8	2.7	58.4	Weight loss	–3.8
3	3.1	3.3	4	47.6	Weight loss	–2.4
4	...	4.4	5.5	28.7	Asymptomatic	+3.4
5	7.1	11.2	16.6	22.9	Weight loss; intermittent symptoms^f^	–12.8
6	5.0	4.9	8.3	33.8	Weight loss	–4.0
7	5.3	5.8	8.3	25.5	Weight loss	–4.3
8	...	14.9	16.6	17.8	Asymptomatic	+6.3
9	2.3	1.9	2.8	22.9	Weight loss	–2.1
10	...	0	...	43.3	Asymptomatic	...
11	1.3	2.8	5.6	7.6	Weight loss	–0.6

Abbreviation: TB, tuberculosis.

^a^TB recurrence date is the date of first bacteriologic confirmation of TB.

^b^Inclusive of date of sputum positivity to date of reversion to sputum negativity.

^c^Smear-negative and culture-positive on diagnosis of TB recurrence with post-treatment smear and culture reversion seen in participants 1–4, 6–9, and 11. No follow-up smear or culture results available for participant 3. Participant 5 was smear- and culture-positive upon diagnosis with smear and culture reversion after treatment initiation. Participant 10 was smear-positive and culture-negative on diagnosis of TB recurrence with no available culture results after treatment start.

^d^Radiologic features: no abnormality detected in 9 of 11 subclinical TB participants on treatment. Participants 4 and 11 presented with bilateral pulmonary infiltrates only.

^e^From diagnosis of TB recurrence to month 12 post-recurrence.

^f^Participant 5 also experienced loss of appetite, unexplained persistent cough, and mild fever intermittently over 3 months.

**Table 3.** Subclinical and Clinical Tuberculosis Recurrence With Spontaneous Resolution

Participant^a^	Months to Symptom Onset from Bacteriologic Diagnosis of Recurrent TB	Duration Between Last Recent TB Outcome and Recurrent TB Episode, (Months)^b^	Time to culture conversion from recurrent TB diagnosis (Months)^c^	Months on Antiretroviral Therapy at TB Recurrence	Gender	Age, Years	CD4 + Count at TB Recurrence, Cells/mm^3^	Clinical Features of TB Disease Progression	Relative Change in Weight,^d^ %
Subclinical TB^e^									
1	...	62.8	5.5	67.4	M	40	369	Asymptomatic	0
2	8	59.2	2.9	63.5	F	33	1046	Weight loss	–0.9
3	...	32.9	2.7	37.2	F	26	846	Asymptomatic	+9.3
4	...	40.0	... ^f^	39.4	M	37	405	Weight loss	–6.4
5	3	33.7	3.0	39.4	F	53	679	Weight loss	–2.8
6	1	36.4	13	39.6	F	38	533	Weight loss	–2.4
Clinical TB^g^									
1	...	65.0	2.8	68.8	F	29	581	Symptomatic	–7.4
2	...	57.6	...	63.5	M	37	498	Symptomatic	+3.1

Abbreviations: CD, cluster of differentiation; TB, tuberculosis.

^a^Viral load (copies/mL) at TB recurrence was undetectable for all participants with spontaneous resolution.

^b^TB recurrence date is the date of first bacteriologic confirmation of TB.

^c^Inclusive of date of sputum positivity to date of reversion to sputum negativity.

^d^From TB recurrence diagnosis to month 12 post-recurrence.

^e^Among subclinical TB participants: participants 1–4 and 6 were smear-negative and culture-positive on diagnosis. Participant 5 was smear- and culture-positive on diagnosis.

^f^Participant 4 was documented as resolved, but no test results were available post-recurrence. The remaining 5 subclinical TB participants reverted to smear- and culture-negative on follow-up.

^g^Among clinical TB participants: participant 2 was smear-negative and culture-positive on diagnosis. No test results were available thereafter, but participant remained in follow-up.

### Bacteriologic Testing for *M.tb* in Subclinical TB Cases and Progression to Clinical TB

Among subclinical TB participants, 82.3% (14 of 17) were sputum smear–negative and culture-positive (Tables 2 and 3, Supplementary Figure 2). Among 16 culture-positive subclinical TB patients, 9 were culture-positive on both liquid and solid media and 7 were culture-positive in liquid media only, with colony-forming unit (CFU) count on solid media ≥10 in 5 subclinical TB participants (Table 1). Among the 27 of 31 clinical TB participants with culture results, 7 were culture-positive on liquid media only and 9 were culture-positive for *M.tb* on both liquid and solid media, with CFU count ≥10 in 8 participants (Table 1). All subclinical TB participants reverted to negative sputum smear and culture, irrespective of TB treatment initiation. Weight loss, heralding TB progression, was observed in 64.7% (11 of 17) of subclinical TB participants with weight loss of <5% in 9 of 17 participants, 5%–10% in 1 of 17 participants, and >10% in 1 of 17 participants (Tables 2 and 3).

### TB Treatment Outcomes

Overall, 83.3% (40 of 48) of subclinical and clinical TB participants initiated anti-TB therapy (Supplementary Figure 2), 64.7% (11 of 17) in the subclinical TB group (Table 2) and 93.5% (29 of 31) in the clinical TB group. Favorable treatment outcomes were observed in 65.0% (26 of 40) of participants overall, 72.7% (8 of 11) in subclinical TB participants and 62.0% (18 of 29) in clinical TB participants. There were 3 deaths in this cohort, all among participants with clinical TB.

## Spontaneous Resolution of Recurrent TB

All 8 participants who did not start TB treatment experienced spontaneous resolution; 6 in the subclinical TB group and 2 in the clinical TB group (Table 3). Median time to culture conversion was 3 months for subclinical TB participants and 2.8 months for the 1 participant who had clinical TB. Among participants who experienced spontaneous resolution, 1 subclinical TB participant and 1 clinical TB participant did not have post-TB recurrence culture results but remained in follow-up with documented clinical and radiological resolution (Table 3).

Isoniazid preventive therapy (IPT) was initiated in 64.7% (11 of 17) of subclinical TB participants, among whom 10 received IPT after recurrence and only 1 received IPT 232 days before recurrence.

### Subclinical TB Resistance

Overall, 18.8% (9 of 48; 6 clinical TB and 3 subclinical TB) of cases developed drug-resistant TB (DR TB). Of the 3 subclinical TB participants with DR TB, 2 had multidrug-resistant TB and 1 was resistant to other drugs.

## DISCUSSION

In this study, we are among the first to describe similar incidence rates of subclinical and clinical TB at 0.9 (95% CI: .5–1.4) and 1.5 (95% CI: 1.1–2.2) per 100 person-years in vulnerable participants at high risk for developing TB in endemic settings. Despite the small sample size, these findings highlight the need to reconsider the current approach of symptom-based screening for TB testing triage.

Quantifying the burden of subclinical TB has thus far been restricted to post-mortem studies, surveillance data, and cross-sectional studies, which accounts for the lack of reliable subclinical TB incidence estimates. Published reports describe variable subclinical TB prevalence rates in ART-naive PLWHA ranging from 1.0% to 4.3%, while data from ART-experienced participants remain scarce [7, 15, 18, 19]. Surveys conducted between 1990 and 2019 describe asymptomatic TB prevalence rates of 56.4% in Asia and 49.4% in Africa, irrespective of HIV status [6]. A recent South African National TB Prevalence survey identified 2.6% (234 of 9066) bacteriologically confirmed TB cases among surveyed participants who screened positive for TB based on compatible symptoms and/or radiologic abnormality, of whom 57.8% (135 of 234) met the subclinical TB case definition [20]. Recent findings demonstrate that more than two-thirds of culture-positive TB occurred in asymptomatic individuals, far higher than rates reported in the South African National TB Prevalence survey. Furthermore, the proportion of culture-positive asymptomatic TB was 1% among clinic attendees compared with 0.6% among community members. This study concluded that symptom-based TB screening will miss many people with clinical TB, irrespective of TB screening in clinics or community settings [21]. The paucity of estimates for subclinical TB prevalence is not surprising given that prevailing clinical guidelines recommend a positive TB symptom screen for entry into the TB and TPT care cascades. Likewise, routine sputum culture testing for TB is not mandatory in asymptomatic patients, which contributes to our failure to identify a substantial number of persons with subclinical TB. A distinct discernable subclinical phase was not detected among two-thirds of participants with bacteriologically confirmed symptomatic clinical TB in this study. In contrast, time to symptom onset from recurrent TB diagnosis to clinical TB ranged from 1 to 8 months among those with subclinical TB. Subtle weight loss was the only clinical symptom of progression in most subclinical TB patients. This finding of discrete clinical and subclinical TB cases disrupts the prevailing assumption that all cases of clinical TB progress through a subclinical TB phase. Furthermore, we found no discernable differences in demographic, clinical, or laboratory parameters among those with subclinical TB compared with those with clinical TB. The lack of baseline differences undermined our ability to identify risk factors for subclinical TB progression to clinical TB. Available published literature does not fully characterize the natural history of subclinical TB.

Almost no empiric data exist that describe duration and rates of progression of subclinical to clinical TB, including comparisons of prevalence and progression rates of subclinical to clinical TB in immune-suppressed vs immune-competent individuals. To date, prevalence rates of subclinical TB have arisen from active TB surveillance and cross-sectional studies conducted mainly among HIV-immunosuppressed populations. These studies suggest that progression from subclinical TB to clinical symptomatic is more rapid in immunosuppressed individuals such as those living with HIV [7, 22–24]. Progression of subclinical to clinical TB has been reported to occur over 3 days to 2 months in approximately two-thirds of immune-suppressed individuals; this may more likely be due to TB unmasking rather than true subclinical TB progression [7, 22, 24]. No data exist that extrapolate similarities and differences of these rates of progression to the general community. Furthermore, published studies restricted to pre-ART cohorts have not shown an association of distinct CD4 count thresholds with prevalence and progression of subclinical to clinical TB [7, 22, 24]. Empiric data describing progression duration and outcomes of subclinical TB in patients living with HIV irrespective of ART status are required to better understand TB pathogenesis and to design interventions to halt progression. Despite a small sample size, observations of impaired CD4 and CD8 function preceding progression from subclinical to clinical TB warrants further evaluation. Additionally, we describe spontaneous subclinical TB resolution in 35.3% of bacteriologically confirmed participants, suggesting that disease resolution is possible in PLWHA. Evidence from the preantibiotic era shows that resolution of TB without treatment in 50% of highly symptomatic participants suggests that spontaneous TB resolution is not rare [25]. Previous studies also postulated that TB cycles through incipient and subclinical TB phases [25]. We provide empiric evidence that supports cyclical activity within TB phases where some participants pass a threshold and progress to clinical TB after the subclinical phase, while others revert to and remain within the incipient TB phase. This dynamic process requires further elucidation.

Smear-negative culture-positive TB accounted for 82.3% of all subclinical TB cases. These participants remained persistently AFB smear-negative and would go undetected in a programmatic setting given the absence of symptoms and bacteriologic TB culture results. Compared with clinical TB, sputum AFB smear positivity in only 17.6% of subclinical TB cases correlated with minimal radiologic findings and lower bacillary loads [26, 27]. However, time to sputum culture positivity among subclinical and clinical TB participants was similar, potentially reflecting similar bacterial burden and risk for onward transmission despite different TB disease phases [22, 28]. Thus, subclinical TB participants who harbor viable TB bacilli may represent an important source of community TB transmission [29, 30].

While it is known that AFB smear positivity correlates with lung bacillary burden with important implications for onward transmission, the implication of persistent culture positivity and possible undetected intermittent sputum *M.tb* shedding on transmission risk remains poorly understood [26, 31]. The likelihood of onward transmission from paucibacillary asymptomatic TB is low [11]. Despite this, the risk of transmission from subclinical TB is associated with the degree and duration of infectiousness and availability of susceptible contacts [5]. This transmission risk is further augmented by duration of the subclinical TB phase [11]. Frontline clinicians in TB-endemic settings often do not initiate TB treatment among stable asymptomatic bacteriologically confirmed TB patients. These healthcare professionals experience difficulty in deciding whether bacteriologic evidence of TB in largely asymptomatic patients warrants treatment and whether these patients require the full TB treatment regimen or TPT [32]. Sputum positivity often arises from laboratory contamination or, as documented in children, may represent transient *M.tb* shedding rather than an incident *M.tb* infection [33, 34]. Contamination that led to an erroneous false-positive TB diagnosis was possible in only 1 subclinical TB participant. While our finding of TB drug resistance in approximately 18.8% of the recurrent TB cohort is not unexpected, we also identified 3 cases of drug-resistant subclinical TB, among whom 2 were multidrug-resistant. This underscores the importance of diagnosing and treating subclinical TB and suggests opportunities for TB drug-resistance acquisition in the subclinical TB state.

Although cough facilitates TB transmission, a population-based TB genomic and epidemiologic study inferred that TB transmission occurred before the onset of any TB symptoms, with one-third of all transmission events occurring during the subclinical TB phase [29]. Furthermore, the relative infectiousness of TB in PLWHA remains controversial. Historical studies show a higher TB transmission risk to close contacts from index cases living with HIV, whereas other studies report little or no risk of TB transmission and acquired TB infection among contacts of TB–HIV-coinfected participants [31, 35]. In our study, similar time to culture positivity among subclinical and clinical TB participants is potentially the only risk factor for TB transmission. Despite this, risk factors known to enhance the likelihood of TB transmission such as cavitary disease, persistent AFB smear positivity, or upper lobe infiltrates were not found in our subclinical TB cohorts.

To interrupt *M.tb* transmission and reduce TB incidence rates, future research into expanded TB case finding strategies needs to be prioritized. Targeted universal TB testing in high-risk groups irrespective of TB symptoms, contact tracing, and TB testing among high-risk contacts of subclinical TB cases warrants evaluation. Investigating the role of whole-genome sequencing and phylogenetic linkages between subclinical TB cases will help identify chains of transmission and TB transmission hot spots and improve our understanding of subclinical TB transmission risk. Furthermore, diagnostic modalities that efficiently identify subclinical TB are critical to treating and preventing TB disease progression. Except for differences in asymptomatic and symptomatic disease presentation, subclinical and clinical TB states are radiologically indistinguishable. The prediction of who resolves TB spontaneously, who progresses from subclinical to clinical TB, and who bypasses the subclinical state to develop clinical TB requires further research.

Despite the strengths of this well characterized longitudinal cohort, we acknowledge several study limitations. First, the limited sample size impaired our ability to adequately explore differences in AFB sputum smear positivity, time to culture positivity, radiologic abnormalities, and duration on ART between subclinical and clinical TB states. The findings of TB prevalence and incidence from this study are generalizable only to stable clinic attendees living with HIV and with a previous TB history, whereas data that describe the prevalence of clinical and subclinical TB by Govender et al are generalizable to clinics and communities in a TB-endemic setting. Furthermore, rates of bacteriologically confirmed asymptomatic TB were identified in 36% of this study compared with 76% in the cross-­sectional study [21]. Second, the lack of paired TB isolate sequence data from first and recurrent episodes undermines our ability to quantify the relative contribution of reinfection vs reactivation of previous TB in subclinical and clinical TB cases. Last, while it is possible that a short subclinical TB phase occurred in those with clinical TB, this was not detected in the quarterly follow-up visit schedule.

## CONCLUSIONS

Among ART-accessing PLWHA with previously treated TB, we found similar incidence rates of clinical and subclinical TB. Our study highlights the cyclical nature of TB disease progression and need for reconsideration of current TB screening and testing guidelines. The challenge of using TB symptoms alone for finding and treating TB cases is demonstrated, emphasizing the importance of targeted universal TB screening and testing among high-risk groups. Furthermore, the importance of close clinical observation, timed periodic TB testing, and treatment among cohorts of PLWHA accessing ART services is emphasized.

## Supplementary Data

Supplementary materials are available at *Clinical Infectious Diseases* online. Consisting of data provided by the authors to benefit the reader, the posted materials are not copyedited and are the sole responsibility of the authors, so questions or comments should be addressed to the corresponding author.

ciac185_suppl_Supplementary_Figures

## Notes

***Acknowledgments*. The** research reported here was supported by the South African Medical Research Council (SAMRC). The authors thank the TB Recurrence Upon TB and HIV treatment (TRuTH) Study participants for their contribution; the study team at the Centre for the AIDS Programme of Research in South Africa, eThekwini clinical research site; and the University of KwaZulu-Natal Nelson R. Mandela School of Medicine, Department of Medical Microbiology, for microbiologic laboratory services.

***Financial support*.** The TRuTH study was supported by the Howard Hughes Medical Institute (grant 55007065), as well as the Centers for Disease Control and Prevention (cooperative agreement UY2G/PS001350-02). Patient care was supported by the KwaZulu-Natal Department of Health and the US President’s Emergency Plan for AIDS Relief. The research reported here was supported by the Strategic Health Innovation partnership, Unit of the Strategic Health Innovation Partnerships Unit of the South African Medical Research Council; a grantee of the Bill & Melinda Gates Foundation through the University of Cape Town; and, in part, by the SAMRC and the US National Institutes of Health (grant 1RO1AL151173-01A1).
