# Supplementary material for: Recurrent Subclinical Tuberculosis Among Antiretroviral Therapy–Accessing Participants: Incidence, Clinical Course, and Outcomes
Source: Clin Infect Dis. 2022 Mar 5;75(9):1628–36. doi: 10.1093/cid/ciac185 (PMC9617574; doi:10.1093/cid/ciac185)
Supplement: ciac185_suppl_Supplementary_Figures [file ciac185_suppl_supplementary_figures.pdf]

Recurrent subclinical tuberculosis among ART accessing participants: Incidence, clinical course, and outcomes

Supplementary Figures

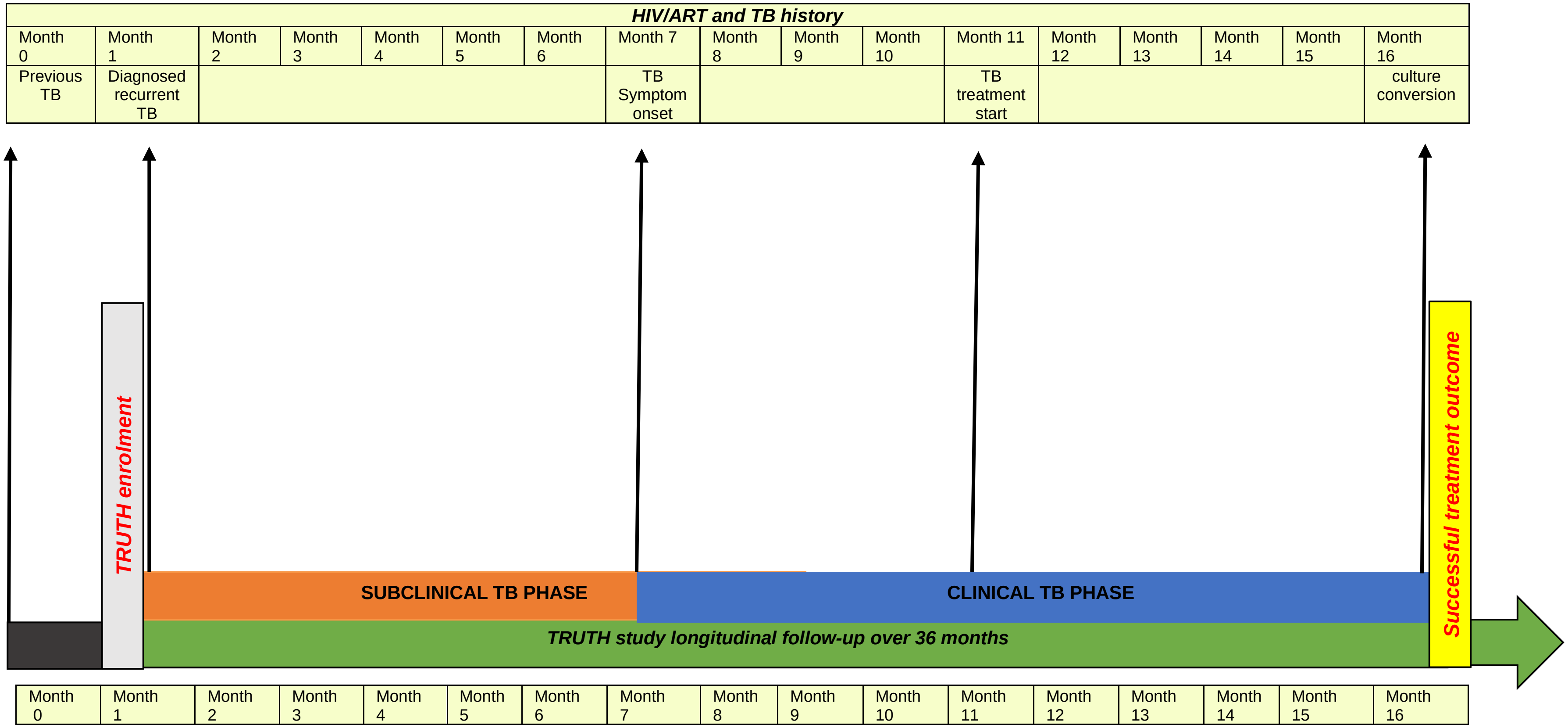

Footnote- Months on ART at recurrent TB: 22.6

Supplementary Figure 1: Longitudinal follow-up of TRUTH study Participant 5 illustrating timeline from previous TB to subclinical TB detection, progression to clinical TB disease and TB treatment outcome

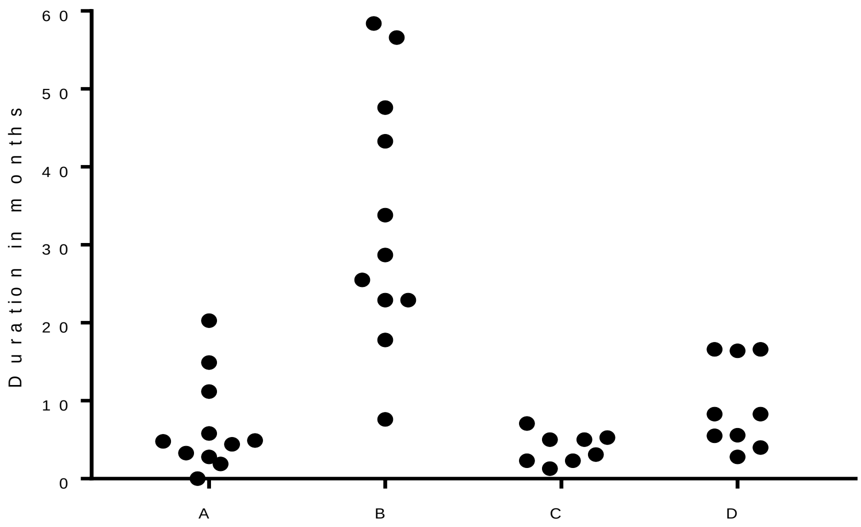

Footnote- Among all subclinical TB participants started on treatment

Supplementary Figure 2: Subclinical TB duration in months from diagnosis to treatment start, time on ART to diagnosis, diagnosis to symptoms, and diagnosis to culture conversion (A: months from diagnosis to treatment start for subclinical TB; B: months on ART at TB recurrence; C: months to symptom onset from time of subclinical TB diagnosis; D: months to culture conversion from TB diagnosis)
